# Enhanced serodiagnostic potential of a fusion molecule consisting of Rv1793, Rv2628 and a truncated Rv2608 of *Mycobacterium tuberculosis*

**DOI:** 10.1371/journal.pone.0258389

**Published:** 2021-11-12

**Authors:** Sadaf Sulman, Saher Shahid, Aasia Khaliq, Atiqa Ambreen, Imran H. Khan, Andrea M. Cooper, Muhammad Waheed Akhtar

**Affiliations:** 1 School of Biological Sciences, University of the Punjab, Lahore, Pakistan; 2 Armed Forces Institute of Pathology, Rawalpindi, Pakistan; 3 Department of Microbiology, Gulab Devi Hospital, Lahore, Pakistan; 4 Department of Pathology and Laboratory Medicine, University of California, Davis, California, United States of America; 5 Department of Respiratory Sciences, University of Leicester, Leicester, United Kingdom; Rutgers Biomedical and Health Sciences, UNITED STATES

## Abstract

Serodiagnosis of tuberculosis (TB) can be rapid, reliable and cost-effective if the issue of variable antibody responses of TB patients against different *Mycobacterium tuberculosis* (*Mtb*) antigens can be overcome by developing fusion proteins containing epitopes from multiple antigens of *Mtb*. In this study, *Mtb* antigens Rv1793, Rv2628, Rv2608 and a truncated variant produced by removing non-epitopic region from N-terminal of Rv2608 (tnRv2608), and the fusion protein Rv1793-Rv2628-tnRv2608 (TriFu64), were expressed in *E*. *coli* and purified. Plasma samples from TB patients characterized by sex, age and sputum/culture positivity, were used to compare the sensitivity of the single antigens with the fusion protein. Sensitivity of Rv1793, Rv2628 and Rv2608, was 27.8%, 39% and 36.3%, respectively. Truncation of Rv2608 increased sensitivity by approximately 35% in confirmed TB cases. Sensitivity of the fusion construct, TriFu64 increased to 66% with a specificity of 100%. Importantly, tnRv2608 was better able to detect sputum and culture negative patients, and this carried through to the fusion protein. We demonstrate that fusion of *Mtb* proteins ensures broad sensitivity across disease types, sex and age groups in a Pakistani population.

## Introduction

Tuberculosis (TB) caused by *Mycobacterium tuberculosis* (*Mtb*) is a global health problem. It is the leading cause of death worldwide by a single infectious agent ranking above HIV/AIDS. In 2019, 10 million people were infected with TB and 1.2 million died globally. About a quarter of world’s population is latently infected with *Mtb*. Among the eight countries accounted for two third of the people infected with TB, Pakistan ranked 5^th^ with 5.7% of total cases. Pakistan accounts for 61% cases in the WHO East Mediterranean regions with 4^th^ highest prevalence of multidrug-resistant TB (MDR-TB). The key factors for drug resistance are delayed diagnosis, unsupervised and inappropriate use of antibiotics, poor follow-up and lack of social programmes [1]. To eliminate TB, the WHO TB End Strategy targets 90% reduction in deaths and 80% reduction in TB incidence annually by 2030 [2]. Two important things are required to eliminate TB. First is the optimized used of current technology to control TB and second is the effective development of a more effective armory of efficient diagnostics, new drugs and more vaccine candidates to replace or augment BCG [3]. Rapid and early diagnosis of TB for control and prevention is crucial to achieve the target [4].

The conventional and current diagnostic tools have several limitations in terms of sensitivity, specificity, efficiency, cost effectiveness and some require sophisticated lab structure which is not feasible in endemic countries like Pakistan [5]. There is unmet need for economical, rapid and biomarker based reliable diagnostic tools that achieve maximum sensitivity and specificity [1, 4]. In this regard, serological methods are inexpensive, prompt and easy to use. Antibodies are a popular candidate as TB biomarkers and a number of *Mtb* antigens have been studied for TB serodiagnosis [6, 7]; the use of single antigens has however failed to achieve reproducible sensitivity and specificity concurrently [4]. Because of the overwhelming benefits of an effective antibody-based diagnostics, it is critical that all approaches are taken to identify antigen combinations that are reproducibly detected by antibodies from a wide range of individuals. To do this we have been studying the ability of multiple *Mtb* antigens combined in unique ways for TB serodiagnostics.

Development of fusion antigens where multiple *Mtb* antigens are combined with a focus on epitope availability may achieve a high level of sensitivity making the serodiagnostic approach practical and economical. We have previously reported different fusion constructs utilizing various antigens from the *Mtb* genome. A truncated variant of PstS1 antigen (tnPstS1) designed by removing non-epitopic regions resulted in enhanced sensitivity from 36% in the native to 43% in the truncated version [8]. A fusion construct, tn2FbpC1-tnPstS1 was developed by joining truncated variants of two antigens FbpC and PstS1 with improved sensitivity of 72.2% [9]. To ensure soluble expression of the fusion proteins the heat shock protein, HspX was added and HspX-tnPstS1 showed 57.7% sensitivity [10]. Rv2031c-Tn1Rv1984c-Rv1352 with the combination of three antigens showed enhanced sensitivity of 71.4% [11] and HspX-EspC-CFP7-PPE57 fusion construct developed by joining four different *Mtb* antigens showed 69% sensitivity [12].

In this study, we have developed a fusion protein using Rv1793, Rv2628 and truncated Rv2608 to produce a Rv1793-Rv2628-tnRv2608 (TriFu64) construct, and we have used ELISA to determine the ability of this construct to sensitively and specifically identify active TB. The selected antigens detected specific antibody responses in a broad range of TB patients and in particular those who are culture and sputum negative. This increased sensitivity holds promise for development of rapid serodiagnostic test for all types of TB.

## Materials and methods

### Study design

Serological samples came from a prospective study, conducted in Gulab Devi Hospital (GDH) and School of Biological Sciences, University of the Punjab (SBS) during a period of December, 2017 to December, 2019. Blood and sputum sample collection, sputum samples’ processing for AFB (Acid Fast Bacillus) smear microscopy and culturing was done at Microbiology department of GDH, whereas blood samples processing and analysis was done at SBS.

### Molecular cloning and expression of the antigens

Antigens included in this study were selected based on published evidence of sero-reactivity in TB [13–15]. The immunodominant T-cell and B-cell epitope sequences of *Rv2608* were obtained from the Immune Epitope Database (IEDB) (https://www.iedb.org) [16]. However, for *Rv1793* and Rv2628 only T-cell epitopes were available, but potential B-cell epitopes of both antigens were predicted through Bepipred-2.0 Sequential B-cell Epitope Predictor (http://www.cbs.dtu.dk/services/BepiPred/), which were found to be overlapping with T-cell antigenic residues [17].

The genomic DNA of *Mtb* H37Rv strain (sourced from National Reference Lab, Islamabad) was used for PCR amplification of full length *Rv1793* (285 bp), *Rv2628* (363 bp) and *Rv2608* (1,743 bp), using their respective primers (Table 1). PCR was done with a 5 min of initial denaturation step at 95°C followed by annealing at 66.7°C for *Rv1793*, 70°C for *Rv2628* and 64.6°C for *Rv2608*, with extension at 72°C for 5 min for 30 cycles and with a final extension was done at 72°C for 20 min.

**Table 1:** List of primers used for PCR amplification of *Mtb* antigens.

Antigens	Primers	Sequences	Restriction sites	Annealing temp. (°C)	Gene length (bp)
*Rv1793*	pF1	GCC**CATATG**ACGATCAATTACCAGTTCGGG	*Nde*I	66.7	285
	pR1	**GAATTC**TTAGGCCCAGTTGGAGCCGAC	*EcoR*I		
*Rv2628*	pF2	ACT**CATATG**TCCACGCAACGACCGAGGCAC	*Nde*I	70	363
	pR2	ATC**GGATCC**TTAGACCGCAACGGCAATCTCAAC	*Bam*HI		
*Rv2608*	pF3	CGT**CATATG**AATTTCGCCGTTTTGCCG	*Nde*I	64.6	1743
	pR3	ATA**GGATCC**TTAGAAAAGTCGGGGTAGCG	*Bam*HI		
*tnRv2608*	pF4	TT**GGCTAG**CGGGAACCTGGGCACG	*Nhe*I	67	1188
	pR4	ATA**GGATCC**TTAGAAAAGTCGGGGTAGCGC	*Bam*HI		
*TriFu64*	pF5	GCC**CATATG**ACGATTAATTACCAGTTCGGG	*Nde*I	66.4	1830
	pR5	ATA**GGATCC**TTAGAAAAGTCGGGGTAGCGC	*Bam*HI		

The truncated variant of Rv2608 (*tnRv2608)* and the fusion construct *Rv1793*-*Rv2628*-*tn2608 (TriFu64)* were also amplified using respective primer pairs using the same PCR conditions with 67°C and 66.4°C annealing temperatures, respectively. Purified *tnPPE42* fragment with *Nhe*I and *Bam*HI restriction sites was ligated into pET28a(+) vector. The double fusion construct *Rv1793*-*Rv2628* carrying *Nde*I and *Nhe*I restriction sites were finally ligated into *tnRv2608* recombinant plasmid to generate the final fusion construct *TriFu64*. The amplified DNA fragments were gel purified, cloned into pTZ57R/T vector and then sub-cloned into a pET28a(+) expression vector, as described previously [10]. The recombinant plasmids were carried by initial cloning host *E*. *coli* DH5α cells.

**Fig 1:**
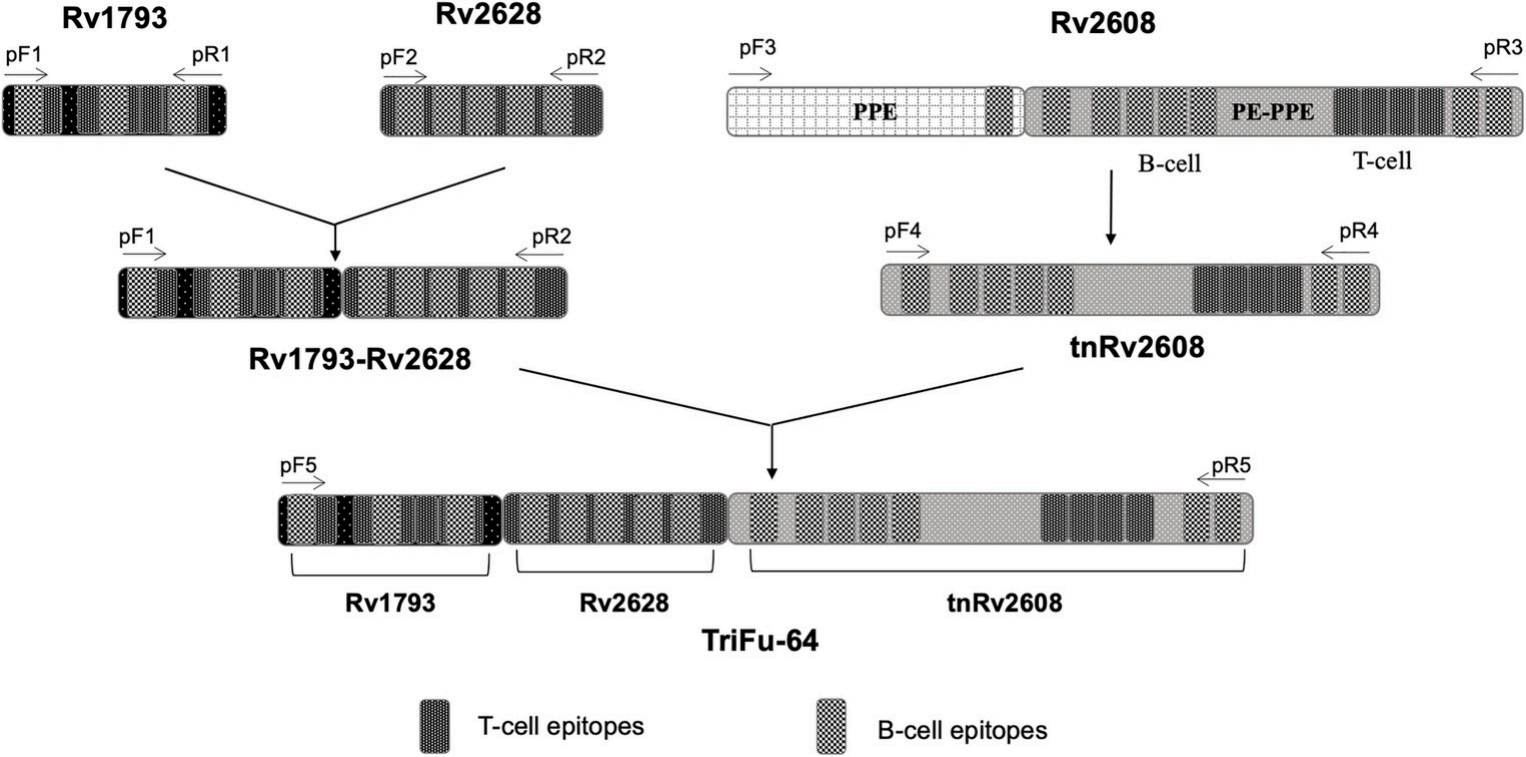
Schematic representation of the epitopes and construction of the single, truncated and fusion *Mtb* antigens. The forward and reverse primers used for each antigen and their constructs are also shown (see Table 1).

The presence and correct orientation of all three gene fragments in fusion construct was confirmed by double digestion of the inserts and sequencing analysis (Apical Scientific Sdn Bhd, Selangor, Malaysia). The schematic diagram for the construction of fusion molecules with their epitopes highlighted is shown in Fig 1.

The expression host *E*. *coli* BL21 Codon Plus was transformed with the recombinant plasmids. The transformed cells were grown in kanamycin (50 μg/ml) supplemented LB medium. The cells expressing Rv1793 were induced with 0.5 mM IPTG grown 0.6–0.8 at OD_600_, at 37°C for overnight, Rv2628 with 0.1 mM IPTG at 37°C for 3 h, Rv2608 with 0.5 mM at 37°C for 1 h, tnRv2608 and TriFu64 were induced with 0.5 mM at 16°C for overnight, according to optimized growth conditions for each of the construct.

### Purification of the antigens

All the proteins were expressed as inclusion bodies. The cells were harvested, washed and the target proteins were purified by Nickel affinity chromatography under denaturing conditions and subsequently refolded as described previously [8]. The total cell protein, soluble, insoluble and purified protein fractions were analyzed on SDS-PAGE and their expression percentage was determined using Syngene gel documentation system (UK). The final protein concentration was estimated by Bradford assay using BSA as standard.

### Evaluation of polyclonal rabbits’ antisera

Polyclonal antisera against the native proteins Rv1793, Rv2628 and Rv2608 were raised in rabbits (*Oryctogalus cuniculus*). Two rabbits, one male and one female, were injected with each of the antigens. The protocol for animal study was approved by Institutional Ethical Committee (SBS/767E/17). All the procedures were performed according to recommended guidelines of Canadian Council on Animal Care (CCAC). The immunoreactivity of proteins Rv1793, Rv2628 and Rv2608 was evaluated against anti-Rv1793, anti-Rv2628 and anti-Rv2608 antibodies by ELISA and Western Blot Analysis according to the method described previously [11].

### Plasma sample collection

This study was approved by Institutional Review Board (IRB No. 0005281) and GDH (GDEC/18-269/17), and was conducted in accordance with the guidelines of the Helsinki declaration for human research. The study objectives were explained to all the participants (TB patients and healthy individuals) in their native language. After obtaining the written consent, the sputum and blood samples were collected along with demographic and clinical data, according to approved standard operating procedures [18].

Sample collection was done from Outdoor Patient Department (OPD) of GDH, Lahore, Pakistan. In GDH, the screening, diagnosis and treatment of TB patients is done according to World Health Organization (WHO)’s guidelines [2]. TB patients were screened through AFB sputum smear microscopy and further subjected to cultures on Lowenstein-Jensen (LJ) and Mycobacterial Growth Indicator Tubes (MGIT) media for confirmation. Out of 400 TB patients, 265 patients were smear or AFB positive/culture positive (AFB^+ve^/Cul^+ve^), 60 patients were AFB negative/culture positive (AFB^-ve^/Cul^+ve^) and 75 patients were AFB negative/culture negative (AFB^-ve^/Cul^-ve^). The AFB^-ve^/Cul^-ve^ patient group was confirmed by clinical follow up, including complete physical examination, chest X-ray (CXR) and response to 6 months anti-TB treatment (ATT). The patients receiving ATT showed positive response to the treatment and substantially improved clinical and radiological findings at the end of ATT, as compared to the time of diagnosis, confirming that they were TB positive. The detailed workflow and protocol of patient selection followed in this study has been described by Khaliq et al. [5].

### Demographic characteristics of TB and HC

A total of 400 pulmonary TB patients (median age 28 y; IQR: 16–80 y), category I (who did not have previous history of TB infection or treatment) were recruited in this study. The study population comprised of both sexes >15 years of age. The prevalence of various symptoms amongst 400 TB patients enrolled in this study were cough (94%), fever (91%), weight loss (84%), loss of appetite (76%) and haemoptysis (27%). Of the enrolled patients 62% were BCG vaccinated and 22% had close contact with active TB patients either in household or at work. Among co-morbidities, 14% TB patients were diabetics and 30% were active-smokers. All the patients were screened for HIV using rapid HIV testing kit (Advance Quality Rapid Anti-HIV (1 & 2) Test Card (whole blood/serum/plasma) by Intec Products Inc. Xiamen, China; Catalog Number: ITP02002). HIV positive active TB patients were excluded from the study.

The blood sample of healthy individuals (n = 141) of both genders (median age 30 y; IQR: 20–50 y) was collected from the same geographical area as that of TB patients. These individuals had no history of active TB, pulmonary symptoms or known medical conditions (infection, cancer, or metabolic disease). All the individuals were BCG vaccinated and tested negative for LTBI as tested by QuantiFERON®-TB Gold assay [19]. The demographic details of TB and HC are listed in Table 2.

**Table 2:** Demographic data of TB patients and healthy controls.

Variable	TB (n = 400)	HC (n = 141)
Age, y (median; IQR)	28;16–80	30; 20–50
Male	245 (61%)	97 (69%)
Female	150 (39%)	44 (31%)
Cough	376 (94%)	13 (9%)
Fever	365 (91%)	4 (3%)
Weight loss	337 (84%)	3 (2%)
Loss of appetite	307 (76%)	2 (1%)
Night sweats	293 (73%)	2 (1%)
Haemoptysis	109 (27%)	0 (0%)
BCG vaccination	248 (62%)	140 (99%)
History of TB contact	87 (22%)	26 (18%)
Diabetes	55 (14%)	13 (9%)
Smoking	117 (30%)	13 (9%)

### Detection of antibodies through ELISA

For detection of human antibodies specific for the proteins in the plasma samples of active TB patients, indirect sandwich ELISA was performed according to the method described previously [8, 11]. The coating concentration for each antigen was 2 μg/ml in 1X PBS buffer (pH 7.4), on 96-well maxisorp microtitre plates (Nunc, Denmark, 4912). The differential absorbance, OD_450/630_ was read with HUMAREADER plus (Human GmBH, Germany). All the plasma samples were tested in duplicate and their average values were calculated for further analysis.

### Statistical analysis

For statistical analysis of ELISA results, the mean and cut-off values of test samples Healthy Controls (HC), n = 141 and TB patients (TB), n = 400) were calculated. The cut-off values of all the antigens were determined by adding standard deviation to the mean OD_450/630_ of the HC (n = 141) samples. OD_450/630_ of each antigen was normalized by dividing by its respective cut-off value. The samples with OD_450/630_ above the cut-off value of 1 were considered positive. The difference between the mean values of TB and HC for each antigen was calculated using One-way ANOVA (non-parametric) with p<0.0001. The sensitivity, specificity, test efficiency (TE), and Mathew Correlation Coefficient (MCC) were calculated. Sensitivity was calculated by dividing the number of positive samples with total number of TB patients. Specificity was calculated by dividing the number of healthy samples (OD_450/630_ below 1) with total number of healthy samples. All the statistical analysis was performed using GraphPad Prism (Version 8, San Diego, CA).

### *In silico* structural analysis

The 3D structures of the proteins were predicted using RaptorX software (http://raptorx.uchicago.edu) [20], a template-based modelling tool. The predicted models were further validated by WHATCHECK and Verify3D (https://saves.mbi.ucla.edu) [21]. The geometric validation of 3D models was performed using the *MolProbity* web service (http://molprobity.biochem.duke.edu) that evaluates model quality through covalent geometry and torsion angle criteria [22]. The solvent accessibility of the amino-acid residues was analysed through the CPORT web server (https://alcazar.science.uu.nl/services/CPORT/) [23] and visualized through Pymol by Schrodinger [24].

## Results

### Expression, refolding and purification of antigens

We selected a panel of antigens from different gene families of the *Mtb* genome, based on their function and reported seroreactivity, these were Rv1793, Rv2628 and Rv2608. Due to the presence of overlapping T-cell and B-cell epitopes, these antigens have potential to mediate both cell-mediated and humoral immune responses [16, 17]. Rv2608 in particular, drives strong humoral immune responses and has high serodiagnostic potential [25]. The PPE domain of Rv2608 located at N-terminal was removed to generate a truncated variant of Rv2608 of 396 amino acid residues.

**Fig 2:**
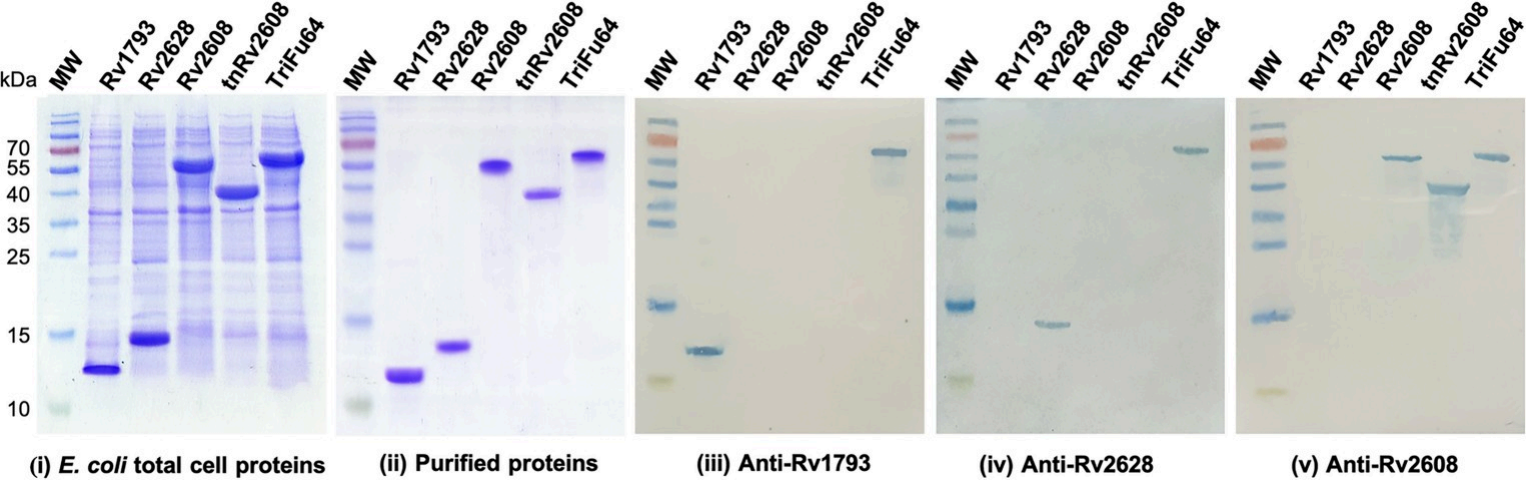
Immunostimulatory activity of *Mtb* antigens produced in *E*. *coli*. SDS-PAGE analysis of the recombinant proteins expressed in *E*. *coli*. (i) Total cell proteins (ii) purified antigens, Western Blots of the purified proteins show the recognition of the individual proteins by rabbit polyclonal antiserum both in the individual and fusion antigens (iii: Anti-Rv1793; iv: Anti-Rv2628 & v: Anti-Rv2608).

Various antigens and their fusion constructs were cloned and expressed in *E*. *coli*. Analysis of the proteins demonstrated expression of each of the various proteins of the expected sizes (Fig 2i). The expression levels of Rv1793, Rv2628, Rv2608, tnRv2608 and Rv1793-Rv2628-tnRv2608 were estimated to be 20%, 28%, 28%, 32% and 36%, respectively. All the proteins were expressed as inclusion bodies, which were purified and refolded. The purified Rv1793, Rv2628, tnRv2608 and TriFu64 were recovered at a rate of 33%, 31.7%, 23.8%, 44.3%, and 27.6%, respectively. Purity level of each of these preparations was above 85% (Fig 2ii).

### Reactivity with polyclonal antisera

**Fig 3:**
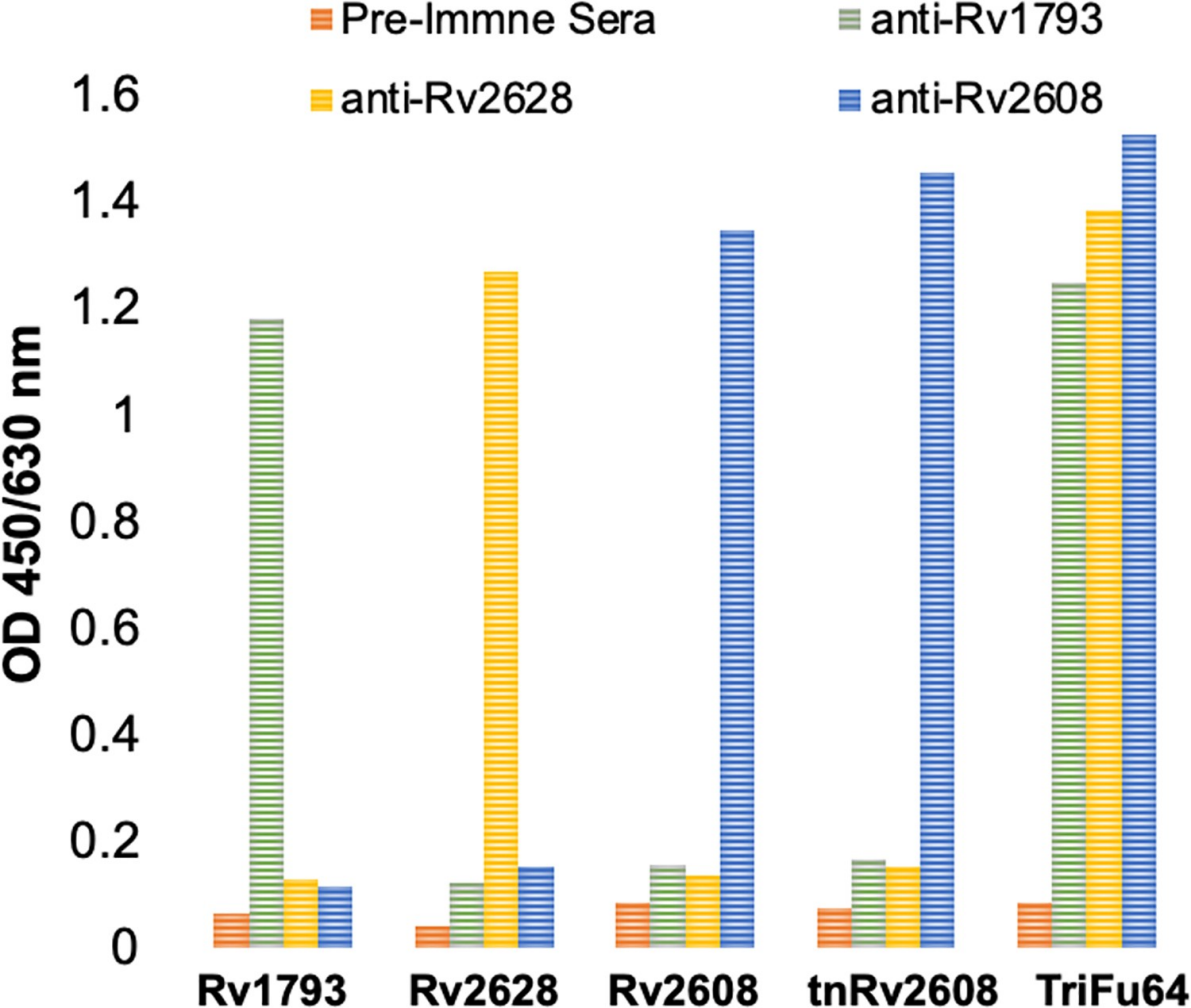
Sero-reactivity of native (Rv1793, Rv2628, Rv2608), truncated (tnRv2608) and fusion (TriFu64) antigens against anti-rabbit polyclonal antisera of native antigens.

The immuno-reactivity of the antigens was determined by immunizing rabbits with the purified proteins. We found that polyclonal anti-Rv1793, anti-Rv2628 and anti-Rv2608 antisera was generated by the rabbits and that this allowed detection of the antigens in both Western Blot analysis (Fig 2iii-2v) and ELISA (Fig 3). The ELISA results indicated significant antibody production in rabbit’s antisera against respective antigens and the fusion protein, TriFu64. Similarly, western blotting indicated anti-Rv1793 reacted with Rv1793 and TriFu64, anti-Rv2628 reacted with Rv2628 and TriFu64, whereas anti-Rv2608 reacted with Rv2608, tnRv2608 and TriFu64 (Fig 2iii-2v).

### ELISA and sensitivity validation

**Fig 4:**
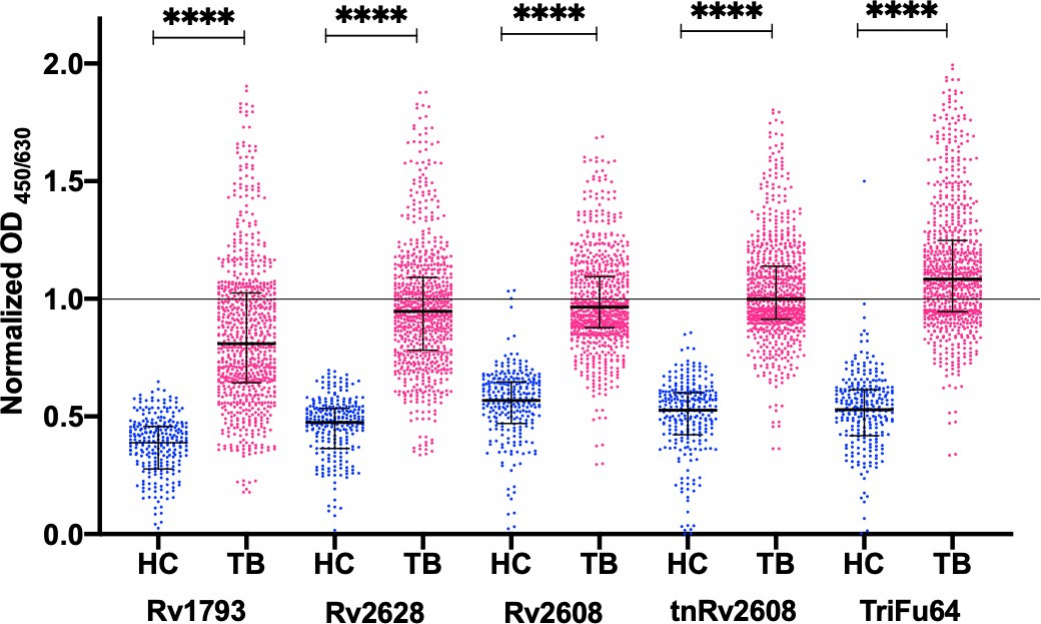
Individual antigens, truncated protein and the fusion protein are differentially recognized by TB patient samples. Scatter plot of normalized OD_450/630_ values showing plasma sero-reactivity of 400 TB patients (TB) and 141 Healthy Controls (HC) against single, truncated and fusion proteins.

ELISA analysis using the proteins as targets and plasma samples from healthy controls (HC) and TB (TB) patients as the probe demonstrated that the individual antigens and the truncated and fusion protein were recognized by the plasma from the TB patients (Fig 4). To confirm there was a difference in the mean OD_450/630_ values of TB and HC plasma samples, non-parametric one-way ANOVA was performed. The mean, median and 95% CI was numerically higher in TB patients as compared to HC with a statistically significant difference (p<0.0001) (Table 3). The mean normalized OD_450/630_ value of TB patients for each antigen was also higher than that of the healthy samples with a statistical difference of p<0.0001(Fig 4).

**Table 3:** Descriptive statistics of one-way ANOVA performed on ELISA results of *Mtb* antigens against healthy controls (HC) and TB patients (TB).

Antigens	Mean OD		Median OD		95% CI				p-value
					lower limit upper limit				
	HC	TB	HC	TB	HC	TB	HC	TB	
**Rv1793**	0.327	0.848	0.388	0.809	0.300	0.827	0.355	0.871	<0.0001
**Rv2628**	0.409	0.962	0.475	0.946	0.382	0.944	0.437	0.980	<0.0001
**Rv2608**	0.517	0.985	0.568	0.965	0.490	0.972	0.545	0.999	<0.0001
**tnRv2608**	0.470	1.036	0.527	0.999	0.443	1.021	0.497	1.051	<0.0001
**TriFu64**	0.483	1.131	0.530	1.083	0.453	1.112	0.512	1.150	<0.0001

Out of the 400 plasma samples, 274 samples were positive for one or more antigens. The sensitivity for Rv1793 was 27.8%, for Rv2628 39%, for Rv2608 36.3%, for tnRv2608 49% and for TriFu64 66%. The specificities of Rv1793, Rv2628 and TriFu64 were 100%, whereas for Rv2608 and tnRv2608, specificities were 98.6% and 99.3%, respectively (Table 4).

**Table 4:** Sensitivity and specificity of *Mtb* antigens against the total plasma samples and the sample groups based on smear and culture results.

Antigens	Total (n = 400)	Sensitivity (%)	Specificity (%)	TE	MCC	AFB^+ve^/Cul^+ve^		AFB^-ve^/Cul^+ve^		AFB^-ve^/Cul^-ve^	
						(n = 265)		(n = 60)		(n = 75)	
						+ve samples	Sensitivity (%)	+ve samples	Sensitivity (%)	+ve samples	Sensitivity (%)
**Rv1793**	111	27.8	100	46.6	30.2	81	30.6	15	25	15	20
**Rv2628**	156	39	100	54.9	37.8	110	41.5	23	38.3	24	32
**Rv2608**	145	36.3	98.6	52.5	34.4	100	37.7	19	31.7	26	34.7
**tnRv2608**	196	49	99.3	62.1	44.1	129	48.7	30	50	38	50.7
**TriFu64**	262	66	100	74.5	57.5	170	64.2	43	70	51	68

**Fig 5:**
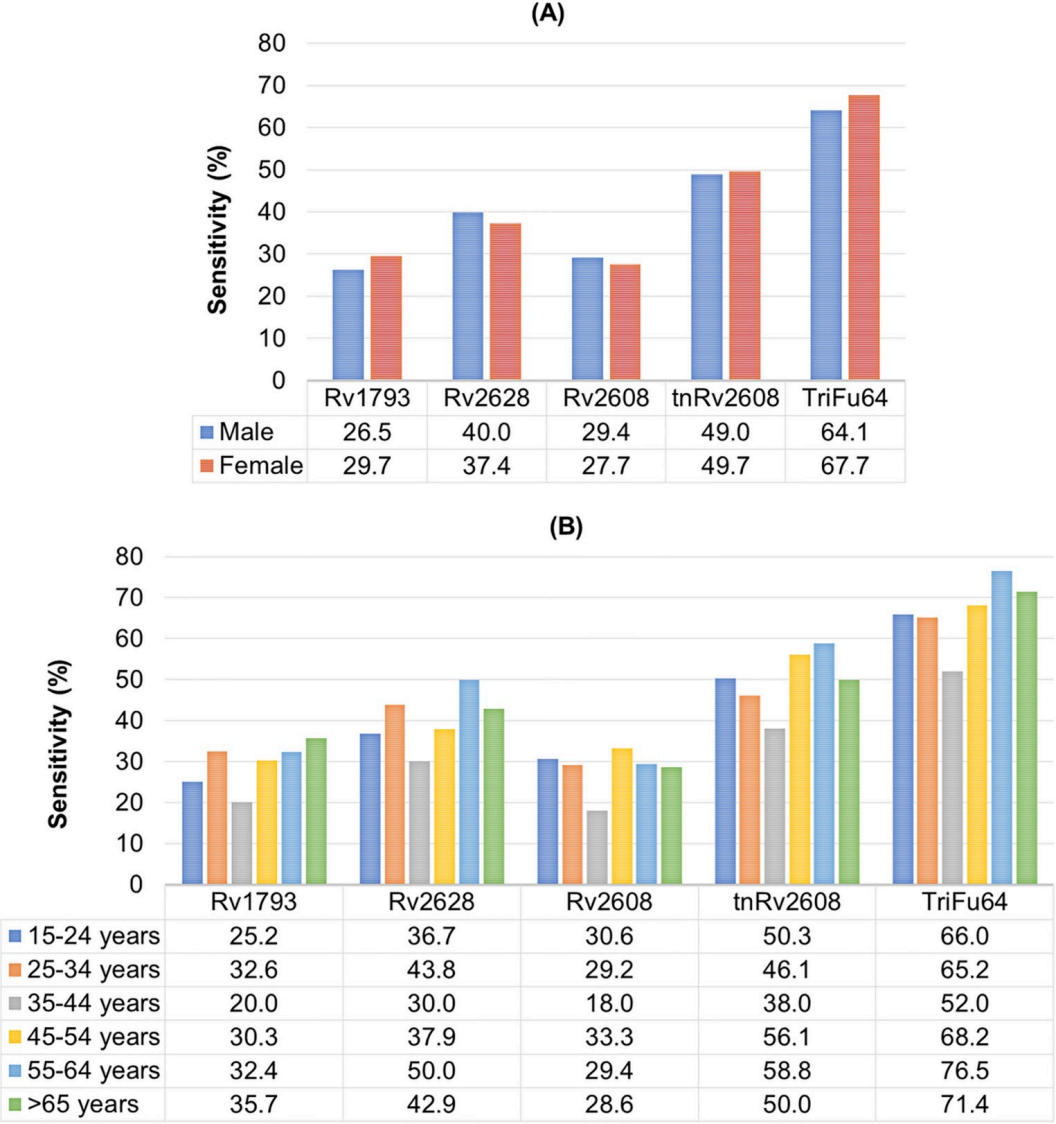
Comparison of sensitivities of single, truncated and fusion antigens among different groups of TB patients by (A) gender groups and (B) age groups.

Since we wanted to understand how the sensitivity of the antigens was impacted by truncation and the fusion within specific groups of patients, we broke the data down by groups (Fig 5A gender, and 5B age). Stratification by gender did not result in broad differences with only a modest increase in females relative to males for the fusion protein (Fig 5A). TB has broad impacts across the age range. We found that truncation of Rv2608 showed significant increase in sensitivity for all age group (Fig 5B) and that the fusion protein was more sensitive than the component antigens. The general pattern of increased sensitivity was similar across age groups however those aged 35-44y appeared to have reduced ability to detect both the component antigens and the fusion protein (Fig 5B)

**Fig 6:**
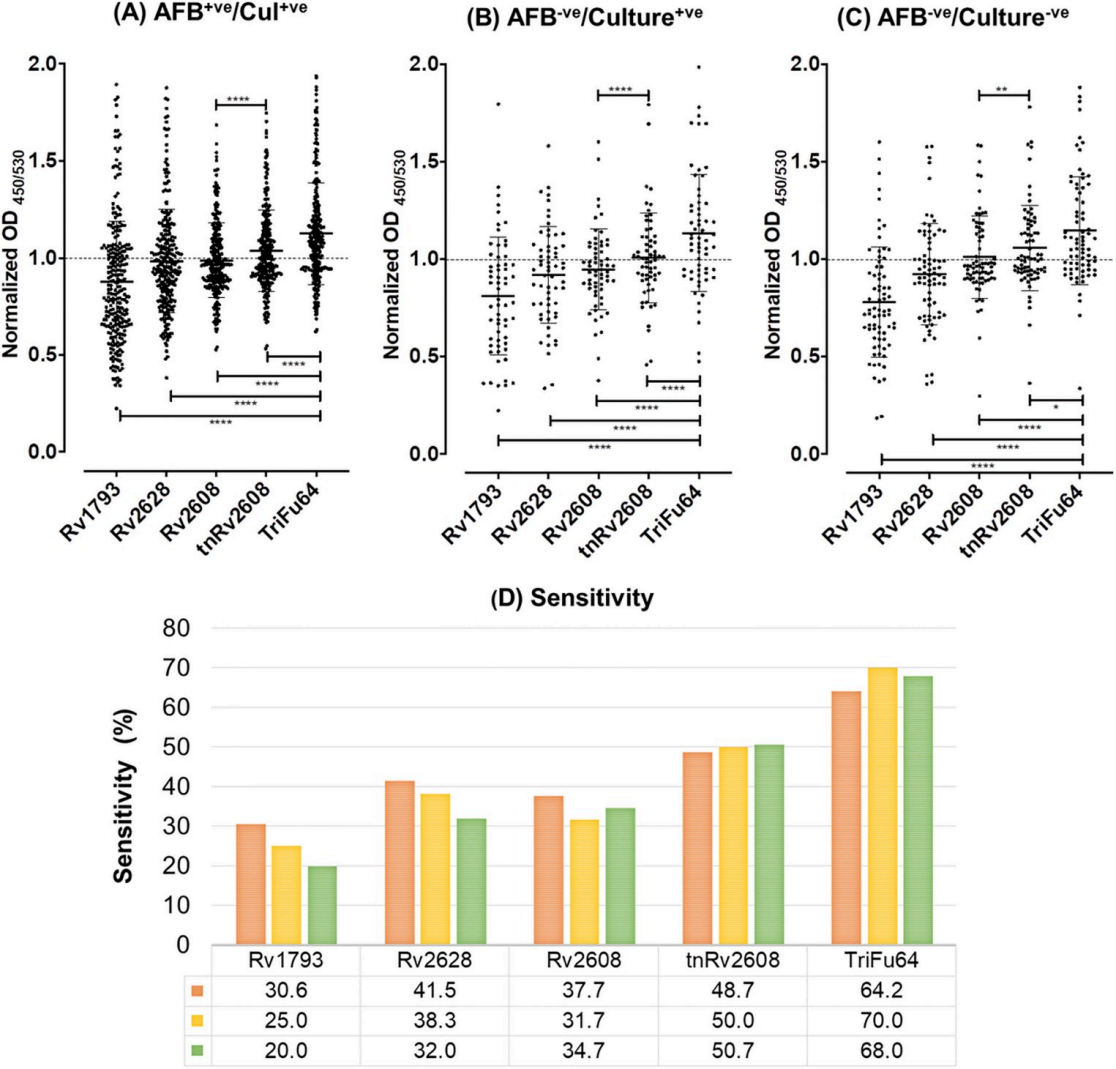
Comparison of sero-reactivity of TB patient groups separated by smear (AFB) and culture positivity. Scatter plot of normalized OD_450/630_ values showing plasma sero-reactivity of AFB positive/Culture positive (A), AFB negative/Culture positive (B), AFB negative/Culture negative (C). The sensitivity of each antigen for each group is also shown (D). One way ANOVA with Dunn’s multiple comparison test ****P<0.0001, **P<0.005, *P<0.05.

Perhaps the most important stratification was by ability to detect bacteria and so we compared the sensitivity to the various antigens within patient groups characterized by smear (AFB) and culture positivity. We found that while Rv1793 and Rv2628 could be detected in patients with smear (AFB) and culture positive (Fig 6A) and smear (AFB) negative and culture positivite (Fig 6B) they performed less well with patients who were both smear (AFB) and culture negative (Fig 6C, Table 4 and Fig 6D). In contrast, both Rv2608 and tnRv2608 were as sensitive in the bacterially negative groups as those with some measure of bacterial presence (Table 4 and Fig 6D). In all groups truncation of Rv2608 resulted in increased recognition by all diagnostic groups (Fig 6A–6C). Critically, the fusion protein showed increased antigen reactivity in all groups (Fig 6A–6C) with equivalent ability to identify the hard to diagnose smear (AFB) and culture negative patients approaching 70% (Table 4 and Fig 6C and 6D).

### 3D molecular modelling

**Fig 7:**
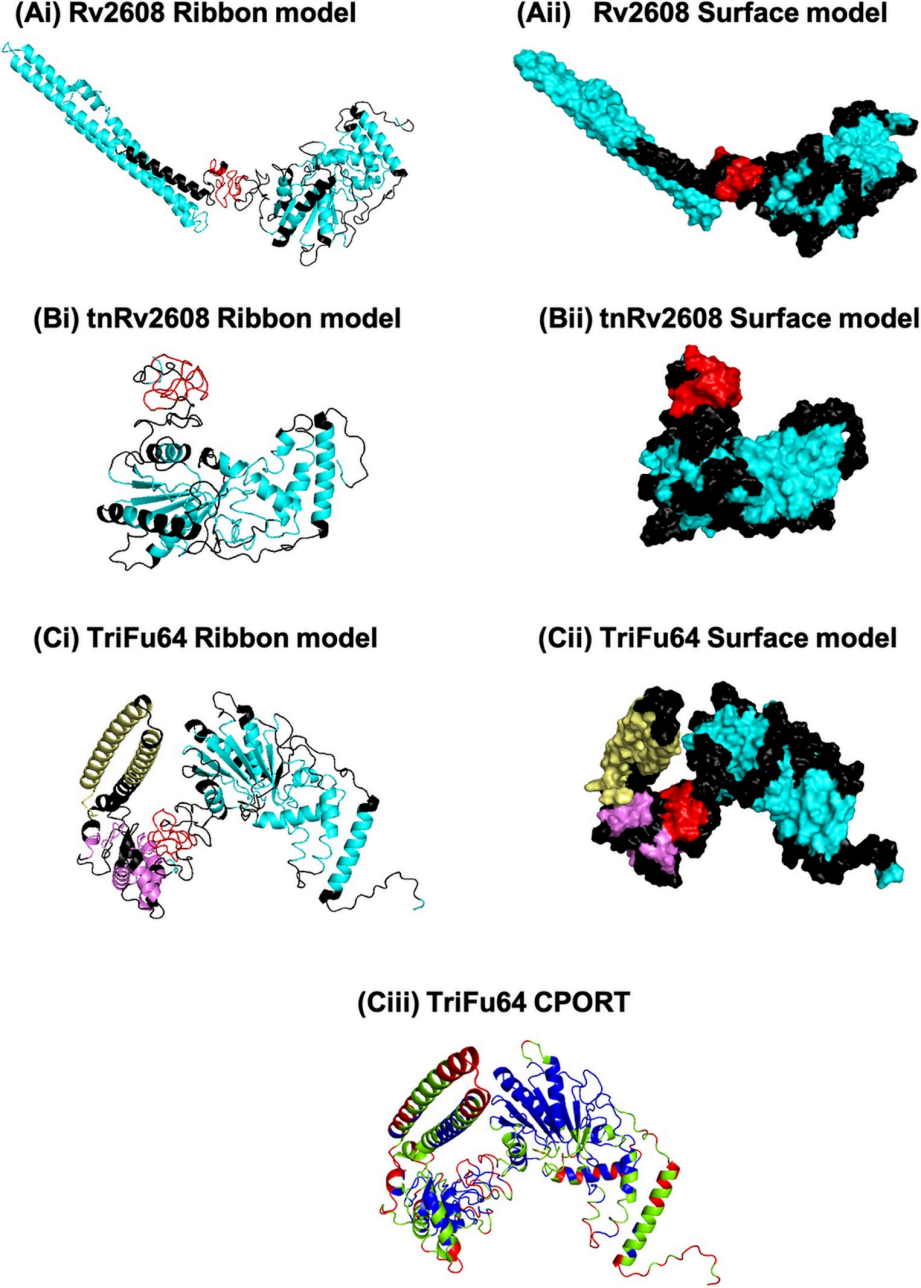
Ribbon and surface models of Rv2608 (Ai and Aii), tnRv2608 (Bi and Bii) and TriFu64 (Ci and Cii). Black color depicts B-cell epitopic regions. Yellow, pink and turquoise depict non-epitopic regions of Rv1793, Rv2628 and tnRv2608, respectively, while the red color indicates the highly antigenic MPTR motif of Rv2608. CPORT analysis structure of TriFu64 (Ciii) shows the solvent accessible (red), the supporting (green) and the non-supporting (blue) residues.

The 3D structural models of all the proteins were successfully generated through RaptorX (Fig 7). All the protein models passed the validation and accuracy tests performed through WHATCHECK, Verify3D and *MolProbity* softwares. In the surface model of Rv2608, the red colour indicates a B-cell epitope that is highly antigenic [25]. This epitope is masked by PPE domain of 184 amino acid residues in the native Rv2608 (Fig 7Aii). However, in the case of the tnRv2608, the antigenic region appears to have been exposed by the truncation and becomes accessible to antibody interaction resulting from the N-terminal domain (Fig 7Bii). The epitopes of native Rv1793 and Rv2628 are significantly solvent accessible. Importantly, when Rv1793 and Rv2628 are attached to the N-terminal of tnRv2608, the B-cell epitope of tnRv2608 remains exposed and accessible suggesting a likely favourable interaction with antibody in the patient’s samples (Fig 7Cii). CPORT analysis of TriFu64 shows that the major epitope regions of Rv1793 and tnRv2608 are red or green, whereas in the case of Rv2628 the epitope regions are red, green and blue (Fig 7Ciii) suggesting that these orientations are not all optimal for accessibility.

## Discussion

Serodiagnosis of TB has high potential, as it can be cost effective and high throughput, if it has an acceptable level of sensitivity and reproducibility. During TB infection, *Mtb* passes through different stages expressing a diverse antigenic profile resulting in a variety of antibody specificities [26]. It has been shown that no single *Mtb* antigen is sufficient to detect the complete antibody profile of active TB patients; therefore, a combination of different *Mtb* antigens should result in improved sensitivity with unaffected specificity [27, 28]. However, combining different antigens in a single polypeptide may create a protein of larger size and there is potential to both accentuate or reduce epitope availability. These potentially disruptive factors can be overcome by generating truncated versions of each antigen and by removing non-epitopic regions which not only decreases the size of fusion antigens but can also increase epitope availability [8, 9, 11].

As a member of PPE family, Rv2608 is unique to *Mtb* [29]. It has conserved N-terminal domain and C-terminal MPTR motif Gly-X-Gly-Asn-X-Gly that induces differential immune responses with a trend towards higher humoral immune responses [25]. Rv2608 is a major polymorphic tandem repeat (PPE_MPTR) subfamily that corresponds to regions of high antigenic index of the protein [29]. When Rv2608 was truncated to tnRv2608 by removing a PPE domain at the N-terminal, it not only reduced the size of protein from 60 kDa to 41 kDa but also resulted in a 35% surge in sensitivity. While designing tnRv2608, the removal of the N-terminal domain exposed a C-terminal MPTR motif at amino acid position 201 to 236 that likely contributed to the enhanced sensitivity. The molecular modelling also supported this hypothesis. Fig 7Aii shows a surface model of Rv2608, in which the MPTR rich motif (colored red) is present at the middle neck of the protein. In contrast, the surface model of tnRv2608 (Fig 7Bii) shows that the MPTR motif has been exposed resulting in enhanced favorable interaction of the antigen with host antibodies. We report enhanced sensitivity (49%) and specificity (100%) of tnRv2608 as compared to the sensitivity (36.3%) and specificity (98.6%) of Rv2608, in the local population.

The addition of low molecular weight antigens such as Rv1793 and Rv2628 at the N-terminal of tnRv2608, allowed the molecular size of the fusion construct to remain low, which eased cloning and facilitated the protein purification process with enhanced stability of TriFu64. We found that TriFu64 was expressed in the form of inclusion bodies and a future development may be the attachment of a heat shock protein (HspX) of *Mtb* at the N-terminal of the construct, which may overcome this as previously shown [12]. Rv1793 (EsxN), which belongs to the ESAT-6 like protein family, is an immunodominant CD4^+^ T-cell antigen capable of inducing cell mediated and humoral immune responses [13, 30]. We report here for the first time that this antigen can be recognized by IgG in human TB plasma samples with 27.8% sensitivity. Rv2628, being one of the 48 co-regulated latency associated antigens of the DosR regulon, induces strong cell mediated and humoral immune responses [14, 31] and is likely expressed later in infection. We report 39% sensitivity of this antigen in active TB cases of the local population.

The fact that the antigens are recognized by human plasma samples by ELISA combined with the *in silico* structural analysis of the TriFu64, suggests favorable and flexible orientation of all the three antigens. This orientation facilitates the interaction of antibodies with the readily available epitopes, enhancing overall sensitivity to 66% (Table 4). Our findings are in accordance with the previous studies where favorable location of antigens enhanced sensitivities to 71.4% and 69% for a triple fusion [11] and the fusion consisting of four antigens [12], respectively.

The CPORT analysis of TriFu64 suggests that major epitopic regions of Rv1793 and tnRv2608 contained actively participating and supporting residues, respectively. However, for Rv2628, the epitopic regions are not only actively participating and supporting residues but some are also non-supporting residues (Fig 7Ciii). The epitopes present in the active and supporting regions of Rv2628 are strong enough to bind with the paratopes of the antibodies. The epitope accessibility of Rv2628 will be a target of future activity for enhanced accessibility by changing its orientation in the fusion construct.

One important population that is difficult to diagnose are those who are sputum (AFB) and culture negative. In this population the truncation of Rv2608 increased sensitivity and by fusing the antigens together, the sensitivity in this population was improved to the point that they were equally well detected as those with apparent bacteria in either sputum or culture, or both. This is a very impressive impact and shows the importance of the fusion protein technique in improving sensitivity for hard to diagnose individuals. This increased sensitivity may reflect the fact that all individuals in this study were BCG vaccinated [32] and that Rv2608 antigen is present in BCG [33]. It may therefore be the case that the local population is sensitized to Rv2608 through BCG vaccination making the antibody response stronger in those cases, thereby increasing sensitivity even in the low bacterial burden group. Comparison of the TriFu64 as a diagnostic tool in non-BCG vaccinated areas might inform this hypothesis. The HC showed quite a high level of reactivity to the *Mtb* antigens in use in this study. TB is endemic in Pakistan and the high reactivity may represent a history of exposure not resulting in symptoms or QuantiFERON®-TB Gold positivity. We have not investigated this further but have used the HC as a baseline to assess the sensitivity and specificity of our protein constructs even in the face of this high background.

Globally, there are more males than females that develop active TB infection [34, 35]. Similarly, in our study, out of 400 TB patients, 61% (n = 245) were male and 39% (n = 155) were female. In contrast to our previous study in which males showed high sensitivity against fusion *Mtb* antigens [11], females showed slightly higher sensitivity for the antigens Rv1793, tnRv2608 and the fusion construct TriFu64 as compared to males [11]. Whether these differences will apply to other populations will need further screening. Interestingly there were variable sensitivities across age groups (IQR: 16–80 y) with the median and 95% CI for each group being statistically different. Importantly, the key groups where rapid diagnosis could make an economic impact are 15–24 y (n = 147) and 25–34 y (n = 89) where we found over 65% sensitivities for both. In order to obtain the desired level of sensitivities further fusion constructs need to be designed and characterized.

## Conclusion

The fusion construct, TriFu64, looks promising for the development of a rapid, efficient and cost-effective TB serodiagnostic, particularly for those pulmonary TB patients who are not identified by culture or smear positivity. Our study also shows sensitivity and specificity in an endemic region with high background reactivity for *Mtb* antigens, which is an important issue for TB diagnostics. This multi-epitope antigen has the potential as a base for further development–possibly with a need to change the orientation of Rv2628 and thereby increasing the level of sensitivity even further for increased serodiagnostic performance.

## Supporting information

S1 File ELISA raw data of TB patients and healthy controls. (XLSX)

S2 File Clinical and demographic data of TB patients and healthy controls. (XLSX)

S1 Raw images Raw SDS PAGE images and raw western blots. (PDF)
