# Supplementary material for: Enhanced serodiagnostic potential of a fusion molecule consisting of Rv1793, Rv2628 and a truncated Rv2608 of Mycobacterium tuberculosis
Source: PLoS One. 2021 Nov 12;16(11):e0258389. doi: 10.1371/journal.pone.0258389 (PMC8589213; doi:10.1371/journal.pone.0258389)
Supplement: S1 Raw images — (PDF) [file pone.0258389.s003.pdf]

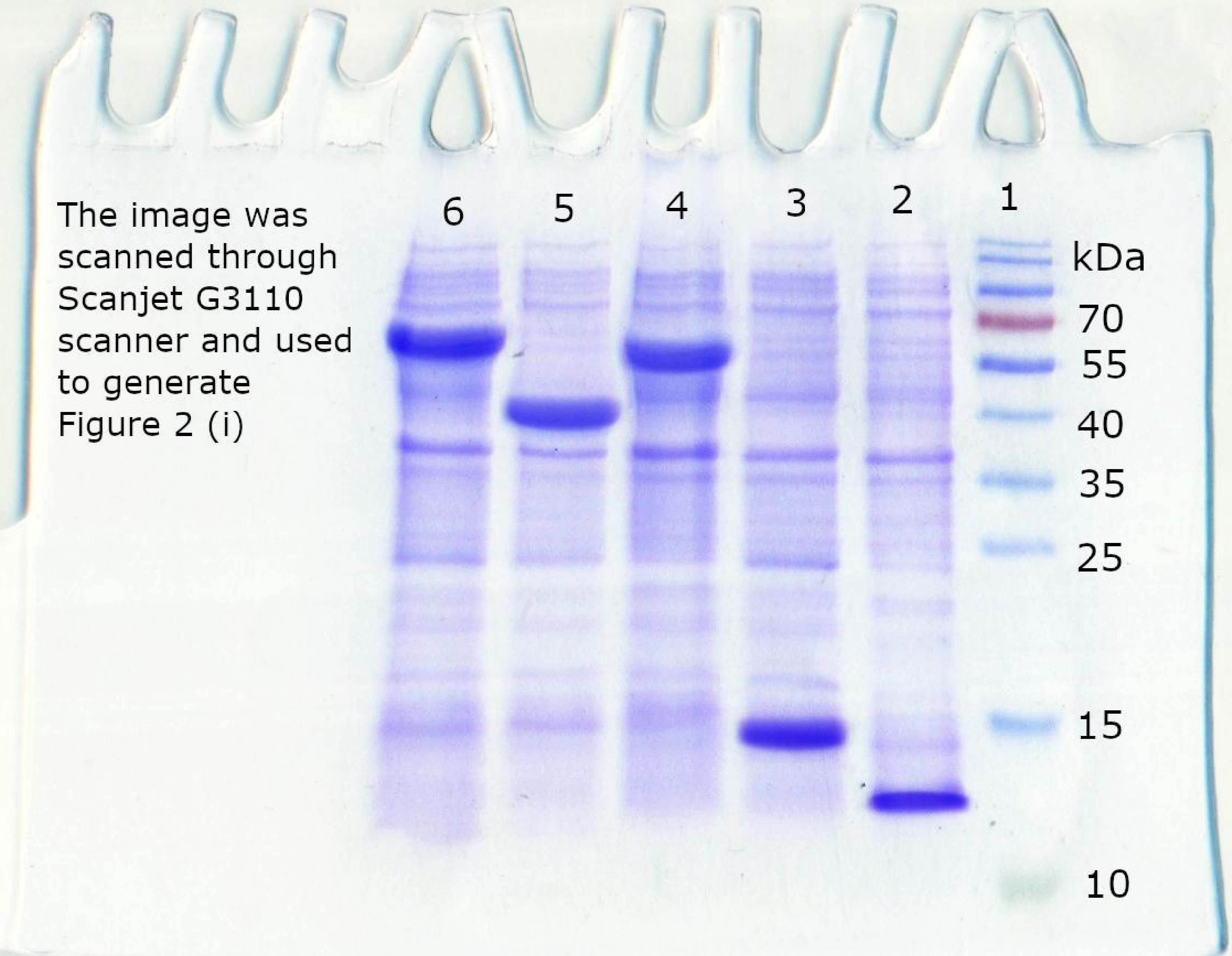

Lane 1: Protein marker; Lane 2: Rv1793; Lane 3: Rv2628; Lane 4: Rv2608; Lane 5: tnRv2608 & Lane 6: TriFu64

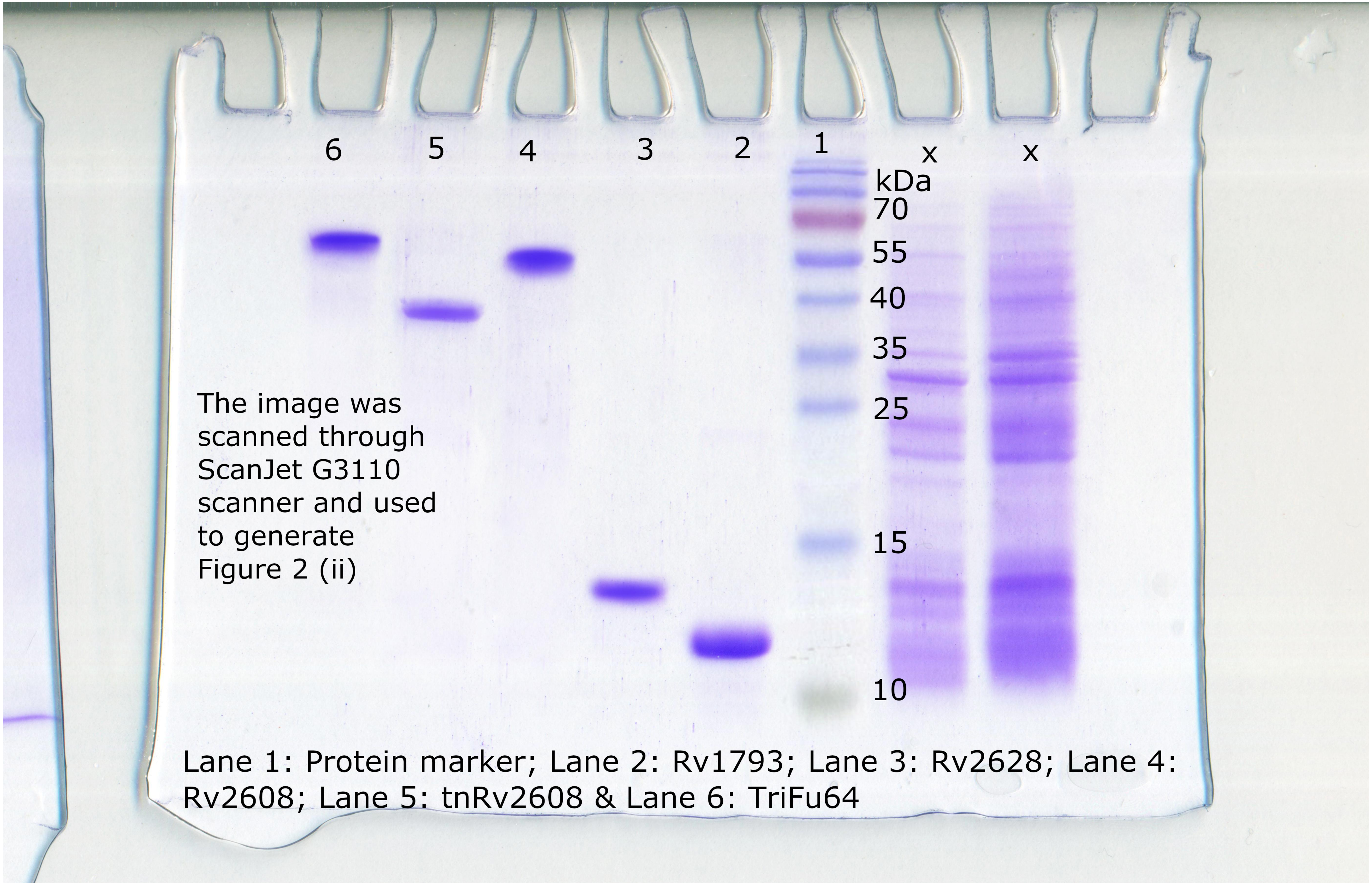
6
5
4
3
2
1
x
x
kDa
70
55
40
35
25
15
10
The image was scanned through ScanJet G3110 scanner and used to generate Figure 2 (ii)
Lane 1: Protein marker; Lane 2: Rv1793; Lane 3: Rv2628; Lane 4: Rv2608; Lane 5: tnRv2608 & Lane 6: TriFu64

The image was captured with Samsung S9 plus mobile camera and used to generate Figure 2 (i)

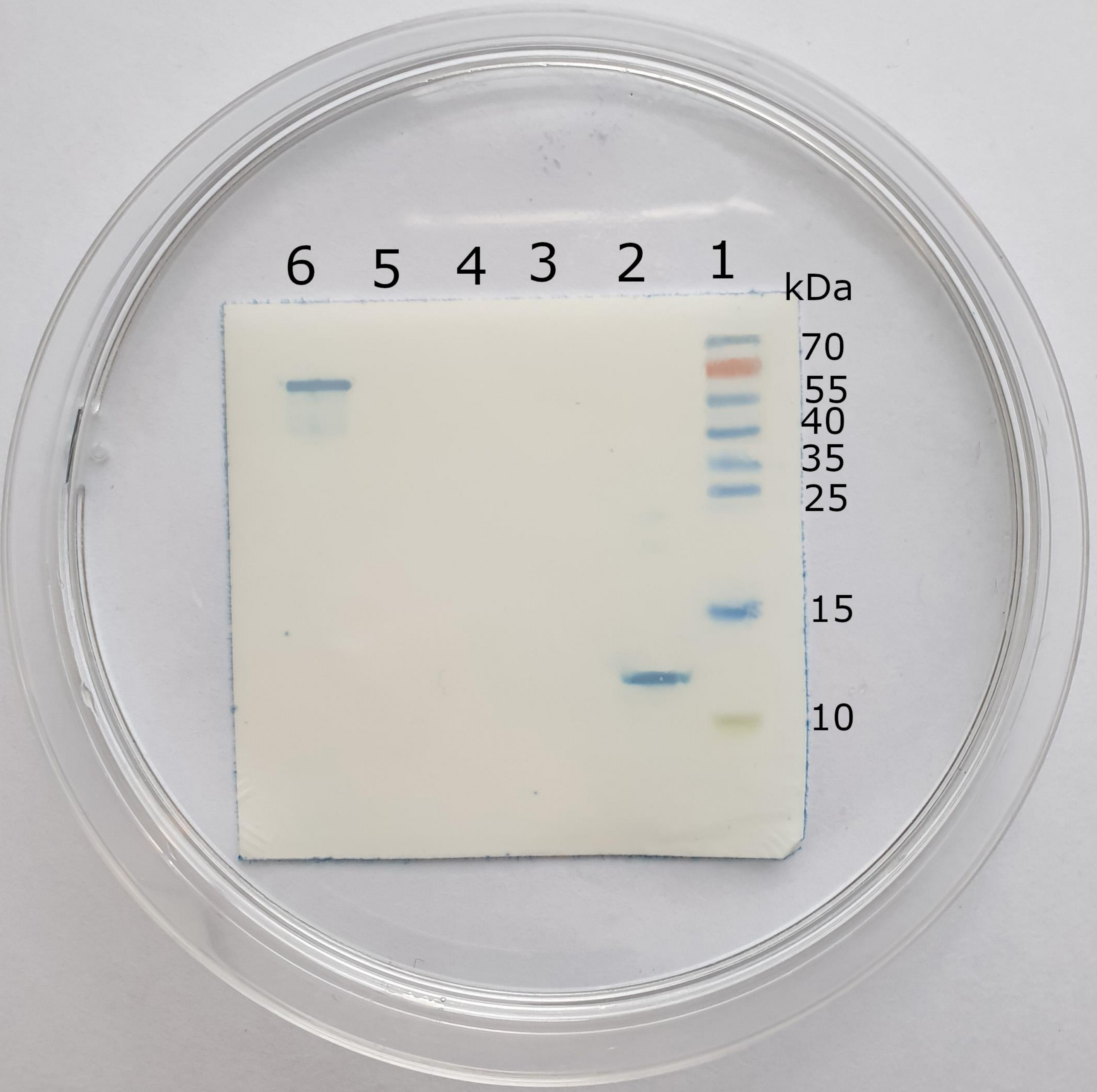

Lane 1: Protein marker; Lane 2: Rv1793; Lane 3: Rv2628; Lane 4: Rv2608; Lane 5: tnRv2608 & Lane 6: TriFu64

The image was captured with Samsung S9 plus mobile camera and used to generate Figure 2 (iv)

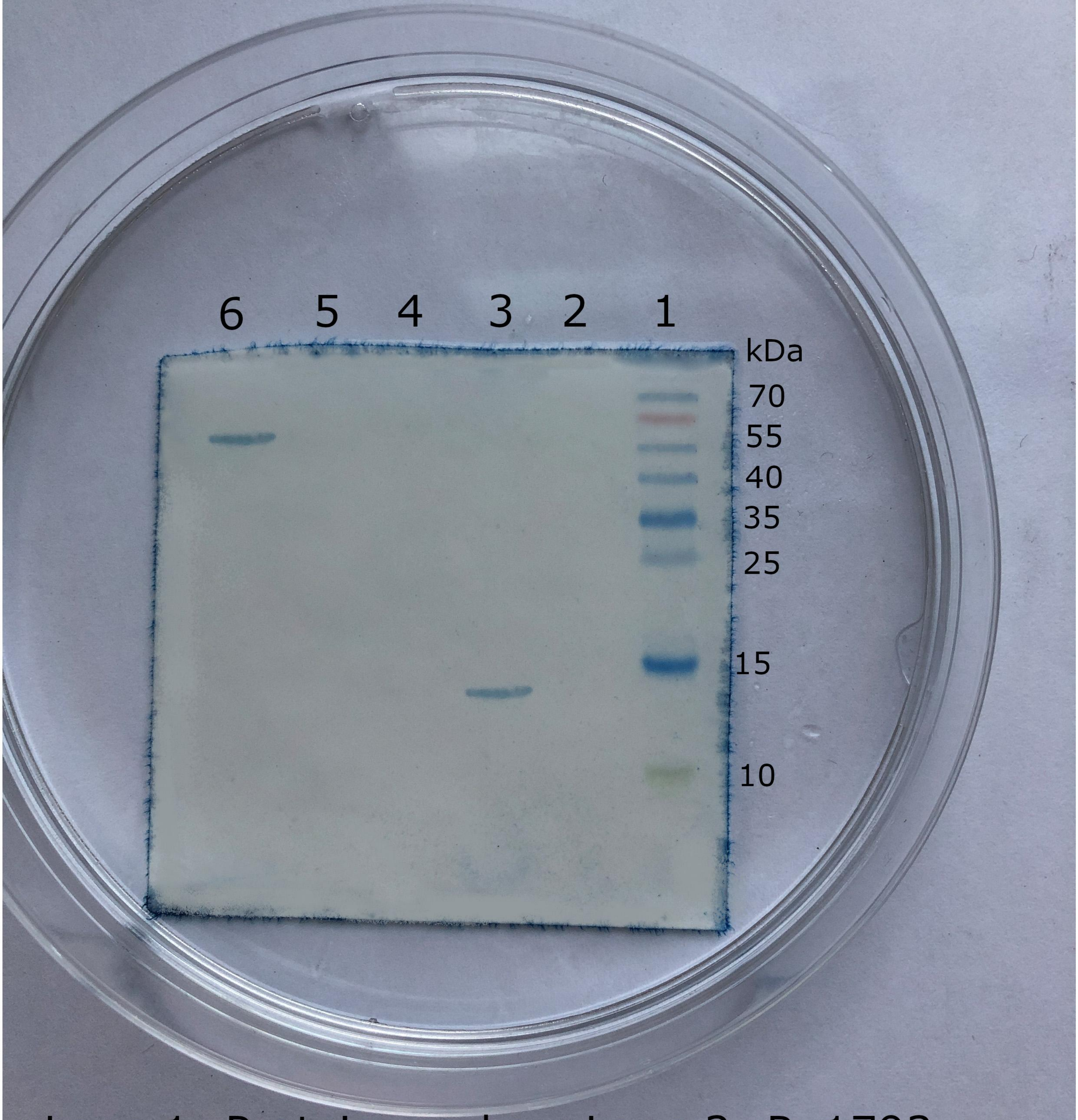

Lane 1: Protein marker; Lane 2: Rv1793; Rv2628; Lane 3: Rv2608; Lane 4: Rv2608 & Lane 6: TriFu64

The image was captured with Samsung S9 Plus mobile camera and used to generate Figure 2 (v)

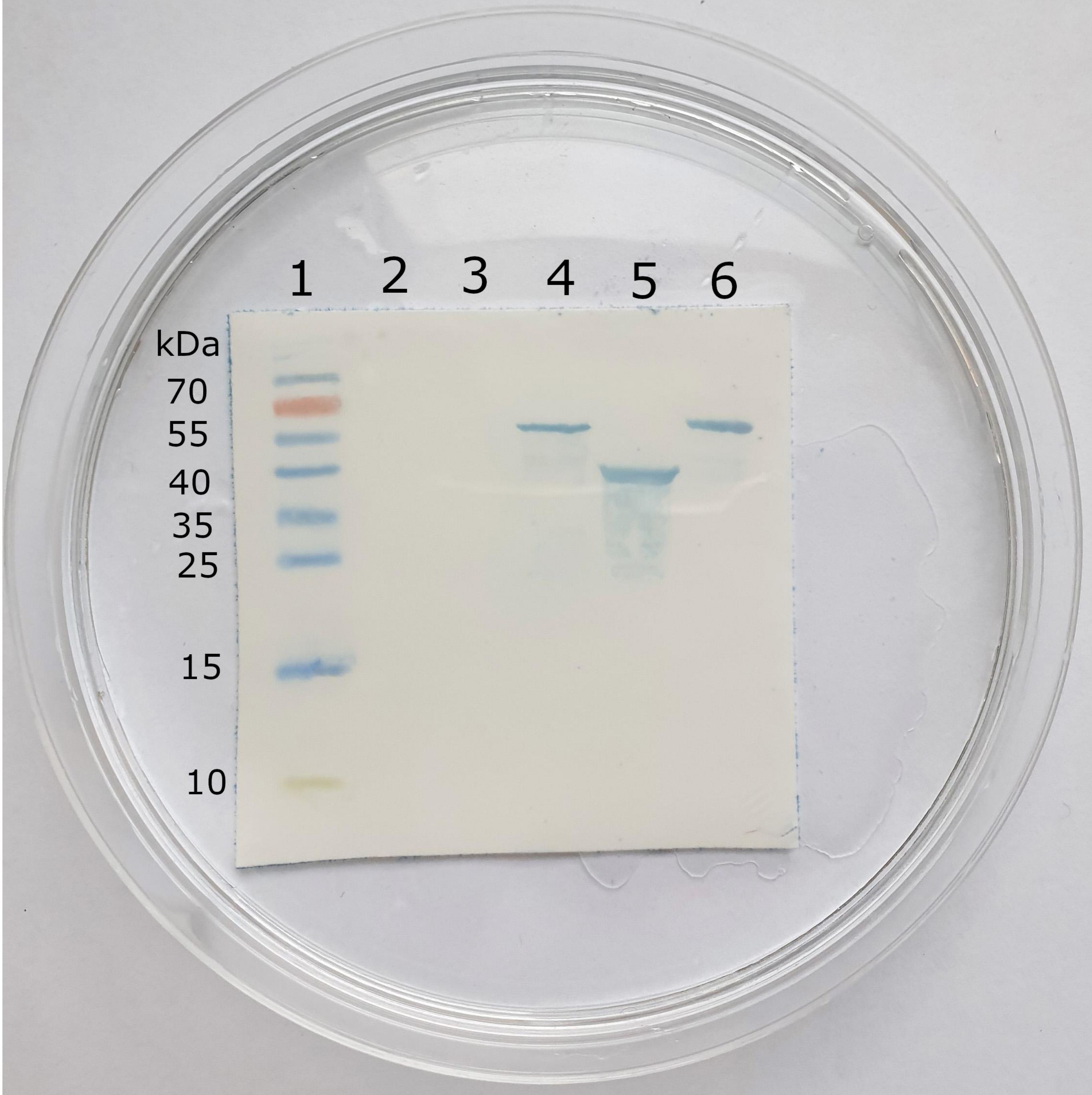

Lane 1: Protein marker; Lane 2: Rv1793; Lane 3: Rv2628; Lane 4: Rv2608; Lane 5: tnRv2608 & Lane 6: TriFu64
